# Sea Ice Detection Based on Differential Delay-Doppler Maps from UK TechDemoSat-1

**DOI:** 10.3390/s17071614

**Published:** 2017-07-12

**Authors:** Yongchao Zhu, Kegen Yu, Jingui Zou, Jens Wickert

**Affiliations:** 1 School of Geodesy and Geomatics and Collaborative Innovation Center for Geospatial Technology, Wuhan University, Wuhan 430079, China; ychzhu@whu.edu.cn (Y.Z.); kgyu@sgg.whu.edu.cn (K.Y.); 2 Key Laboratory of Precise Engineering and Industry Surveying, National Administration of Surveying, Mapping and Geoinformation, Wuhan 430079, China; 3 German Research Centre for Geosciences, GFZ Potsdam 14473, Germany; wickert@gfz-potsdam.de; 4 Institute of Geodesy and Geoinformation Science, Technische Universität Berlin, Berlin 10623, Germany

**Keywords:** sea ice, GNSS-R, Delay-Doppler Map (DDM), differential DDM (dDDM), UK TechDemoSat-1 (TDS-1)

## Abstract

Global Navigation Satellite System (GNSS) signals can be exploited to remotely sense atmosphere and land and ocean surface to retrieve a range of geophysical parameters. This paper proposes two new methods, termed as power-summation of differential Delay-Doppler Maps (PS-D) and pixel-number of differential Delay-Doppler Maps (PN-D), to distinguish between sea ice and sea water using differential Delay-Doppler Maps (dDDMs). PS-D and PN-D make use of power-summation and pixel-number of dDDMs, respectively, to measure the degree of difference between two DDMs so as to determine the transition state (water-water, water-ice, ice-ice and ice-water) and hence ice and water are detected. Moreover, an adaptive incoherent averaging of DDMs is employed to improve the computational efficiency. A large number of DDMs recorded by UK TechDemoSat-1 (TDS-1) over the Arctic region are used to test the proposed sea ice detection methods. Through evaluating against ground-truth measurements from the Ocean Sea Ice SAF, the proposed PS-D and PN-D methods achieve a probability of detection of 99.72% and 99.69% respectively, while the probability of false detection is 0.28% and 0.31% respectively.

## 1. Introduction

Knowledge of sea ice coverage and thickness of the polar region is very important for ship routing [1], oil and gas exploration [2], and global weather studies [3]. Although in situ monitoring may provide continuous sea ice information, the spatial sampling is typically insufficient to provide information on global sea ice extent. Estimating global sea ice coverage with adequate temporal resolution can only be achieved using remote sensing technology.

Global Navigation Satellite System (GNSS) Reflectometry (GNSS-R) has been investigated for many years as an innovative remote sensing technique using GNSS signals reflected off the Earth’s surface [4]. GNSS-R was originally suggested and applied to ocean altimetry in 1993 as a multistatic radar technique [5]. With the availability of GNSS signals from GPS, GLONASS, Galileo and BeiDou satellite constellations, this technique shows great promising. In the following years, applications of GNSS-R were extended to the remote sensing of sea surface roughness [6,7], wind speed and direction retrieval [8,9,10,11], target detection and positioning [12], soil moisture determination [13,14,15,16,17], forest biomass [18,19], oil slick detection [20], tsunami parameter estimation and tsunami detection [21,22], sea surface altimetry [23,24], ocean topography [25,26], coastal and continental water altimetry [27] and snow depth estimation [28]. The potential of GNSS-R Delay-Doppler Maps (DDMs) for sea ice distribution detection is studied in this paper.

GNSS-R-based sea ice detection was first investigated by Komjathy et al. [29] by comparing the waveform peak power of reflected signals over ice obtained from an airborne GNSS receiver with RADARSAT back-scattering echoes. The feasibility of ice sheet sensing using GNSS-R was exploited in [30], which proposed a theoretical model of ice scattering. Then the shape of reflected GPS waveforms was studied in [31] to retrieve the permittivity and roughness of different types of sea ice. The United Kingdom-Disaster Monitoring Constellation (UK-DMC) launched in 2003 provided spaceborne GNSS-R data for sea ice detection for the first time, which captured the coherent reflection component of signals reflected from sea ice [32]. Unfortunately, the relationship between sea ice parameters and waveform peak power has not been established. In addition, airborne and ground-based studies using reflected GNSS signals were also performed to explore sea ice concentration [33]. After the launch of the UK TechDemoSat-1 (TDS-1), a huge number of DDMs are available from the Measurement of Earth Reflected Radio-navigation Signals by Satellite (MERRByS) website at www.merrbys.co.uk [34]. TDS-1 DDM data have been used by many researchers for different applications, including wind speed retrieval [35], ocean wave characteristic inversion [36], Tsunami detection and parameter estimation [37], soil moisture estimation [38], sea surface height estimation [23,39] and sea ice detection [40,41].

The first result of sea ice detection using TDS-1 GNSS-R DDMs was presented in [40], which proposed a pixel number based sea ice detection method. Another sea ice detection algorithm based on the similarity of received reflected waveform or DDM, which performs well on sea ice detection, was proposed by Alonso-Arroyo, et al. [42]. The goal of the paper presented here is to explore a new approach through estimating the degree of difference between adjacent DDMs with the capability of distinguishing between sea ice and water. A new GNSS-R differential DDM (dDDM) observable derived from adjacent DDMs is proposed firstly. Then, two different methods are proposed to characterize the dDDMs, which are power-summation (PS) and pixel-number (PN) based methods. The different transition types (water-water, water-ice, ice-ice and ice-water) can be classified according to the characteristics of dDDMs. Several data processing strategies such as dataset selection, noise floor subtraction, and normalization of DDMs are employed to reduce the impact of noise floor and speckle noise. In addition, an adaptive incoherent averaging technique is employed to improve the computational efficiency. Finally, the performance of the proposed methods is evaluated against the existing methods using a large number of TDS-1 DDMs and ground-truth sea ice edge maps.

The next section of this paper presents the details of the two proposed ice detection methods, including the definition of the new observation variable, the derivation of the formulas, the data processing techniques, and the detection procedures. Section 3 provides the experimental results obtained through processing the TDS-1 DDM dataset using the proposed methods and the existing methods. The final section summarizes the scientific results and indicates future directions for performance enhancement.

## 2. Theory and Method

### 2.1. GNSS-R Delay-Doppler Maps

The TDS-1, launched in July 2014, was placed into a quasi-Sun synchronous orbit with an altitude of ~635 km and an inclination of 98.4°. The Space GNSS Receiver Remote Sensing Instrument (SGR-ReSI) built by Surrey Satellite Technology Ltd. (SSTL, Guildford, UK) is one of the key payloads carried on TDS-1. The ReSI supports the GPS L1 frequency band using a downward pointing antenna with a gain of ~13 dBi. More details about TDS-1 and the SGR-ReSI are introduced in [43].

**Figure 1:**
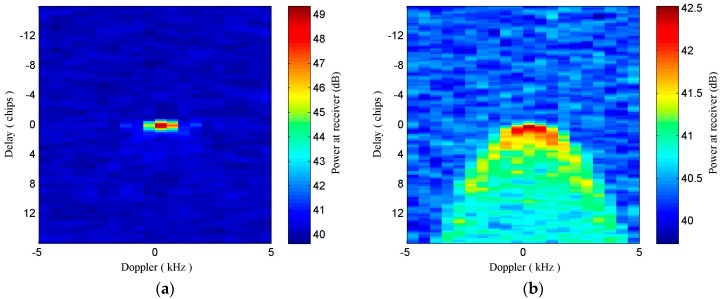
Global Navigation Satellite System Reflectometry (GNSS-R) Delay-Doppler Maps (DDMs) collected over (**a**) sea ice field (close to a specular reflection point) and (**b**) sea water field obtained from the Space GNSS Receiver Remote Sensing Instrument (SGR-ReSI) receiver onboard the TDS-1 satellite on 21 January 2016.

The TDS-1 receiver produces DDMs through cross correlating received scattered signals with locally generated and time-synchronized code replicas for different path delays and Doppler shifts [38]. Speckle noise has a significant influence on cross correlation, which is usually performed over 1 ms and less 20 ms of coherent integration time, so an incoherent summation of consecutive cross correlation values is employed to mitigate the noise. The typical incoherent summation time for TDS-1 DDMs is 1 s [43]. The spreading of DDM reveals the degree of roughness of the observing surface. It is well known that the surface of sea water is usually rougher than that of young ice, so the spreading in Delay and Doppler of DDMs collected over sea ice surface would be less than that of sea water, whose DDMs usually exhibit a horseshoe shape [7]. Typical DDMs from TDS-1 collected over sea ice and water are shown in Figure 1. Figure 1a depicts a DDM collected over an ice field, while a DDM of sea water is shown in Figure 1b, which clearly indicates that the DDM is heavily affected by noise and sea state.

### 2.2. GNSS-R Observables

GNSS-R DDMs are usually processed to produce observables that could describe the scattering strength of reflected surface. Different surface roughness related observables have been proposed in [44,45]. Those DDM observables have been employed in [40] with slight modification, which applied a pixel number based sea ice detection method with an incoherent summation.

The standard DDM observable is usually denoted as DDM(τ,f), where τ and f represent delay and Doppler shift, respectively. Then the corresponding normalized DDM denoted as $\bar{DDM}\left(\tau ,f\right)$ is formulated as:

(1) $$\bar{DDM}\left(\tau ,f\right)=DDM\left(\tau ,f\right)/{\left|DDM\right|}_{\max},$$

where ${\left|DDM\right|}_{\max}$ is the maximum absolute value of the standard DDM.

#### 2.2.1. Forward GNSS-R Observables

One existing observable derived from $\bar{DDM}\left(\tau ,f\right)$ was defined in [45] and later was modified as weighted area in [44], which can be formulated as:

(2) $$Weighted-Area=\underset{\bar{DDM}\left(\tau ,f\right)>\mathrm{thre}shold}{\sum \sum }\bar{DDM}\left(\tau ,f\right)\cdot \delta_{\tau }\cdot \delta_{f},$$

where $\delta_{\tau }$ and $\delta_{f}$ are delay and Doppler shift resolutions respectively. If $\delta_{\tau }\cdot \delta_{f}$ is set as one, this observable represents the normalized power summation of the grids whose $\bar{DDM}\left(\tau ,f\right)$ values are greater than a preset threshold, which is defined through specific processes described in Section 2.4.2.

A similar observable modified from an observable defined in [45] is defined in [40] as the number of grids (i.e., pixel number) whose values greater than a given threshold:

(3) $$Area=\underset{\bar{\mathrm{DDM}}\left(\tau ,f\right)>threshold}{\sum \sum }\delta_{\tau }\cdot \delta_{f}.$$

If $\delta_{\tau }\cdot \delta_{f}$ is set as one, this observable represents the pixel number of $\bar{DDM}\left(\tau ,f\right)$ whose component values are greater than a given threshold, which is defined through specific processes described in Section 2.4.2.

#### 2.2.2. Proposed GNSS-R Observables

In this paper, an observable called dDDM observable is proposed for distinguishing sea ice from water. Details of the proposed observable are provided as follows.

Two adjacent normalized DDM observables can be expressed as $\bar{DDM_{i}}\left(\tau ,f\right)$ and $\bar{DDM_{i+1}}\left(\tau ,f\right)$ respectively. Then the dDDM of these two adjacent normalized DDMs can be simply formulated as,

(4) $${\mathrm{DDM}}_{D}\left(\tau ,f\right)=\bar{DDM_{i}}\left(\tau ,f\right)-\bar{DDM_{i+1}}\left(\tau ,f\right),$$

where ${\mathrm{DDM}}_{D}\left(\tau ,f\right)$ denotes the dDDM. The dDDM can be normalized using Equation (1) and the normalized dDDM is denoted as $\bar{{\mathrm{DDM}}_{D}}\left(\tau ,f\right)$.

Considering that the power value of ${\mathrm{DDM}}_{D}\left(\tau ,f\right)$ can be either positive or negative, two new observables modified from the observable Weighted−Area (Equation (2)) and Area (Equation (3)) (Weighted−Area and Area are math type variables) respectively are defined as,

(5) $$PS=\underset{\left|\bar{DDM_{D}}\left(\tau ,f\right)\right|>DDM_{T}}{\sum \sum }\bar{DDM_{D}}\left(\tau ,f\right)\cdot d\tau \cdot df,$$

(6) $$PN=\underset{\left|\bar{DDM_{D}}\left(\tau ,f\right)\right|>DDM_{T}}{\sum \sum }\frac{\bar{DDM_{D}}\left(\tau ,f\right)}{\left|\bar{DDM_{D}}\left(\tau ,f\right)\right|}d\tau \cdot df,$$

where dτ and df are delay and Doppler shift resolutions for ${\mathrm{DDM}}_{D}\left(\tau ,f\right)$ respectively. If setting dτ⋅df = 1, *PS* represents the power summation and *PN* depicts abstract pixel number of the grids with absolute values ($\left|\bar{{\mathrm{DDM}}_{D}}\left(\tau ,f\right)\right|$) greater than a preset threshold ($DDM_{T}$).

### 2.3. Data Processing

#### 2.3.1. Dataset Selection and Noise Floor Subtraction

As introduced above, a large number of GNSS-R DDMs have been collected over the Arctic region since the launch of TDS-1. The DDMs generated onboard the TDS-1 satellite are available on the data service platform MERRByS, which provides data track view to help select DDM dataset of the area of interest. Since the purpose is to explore methods for distinguishing between sea ice and water, so only DDMs collected over sea ice and water fields are selected for testing. Those datasets acquired over land are excluded with the geographic information obtained from the U.S. National Oceanic and Atmospheric Administration (NOAA).

**Figure 2:**
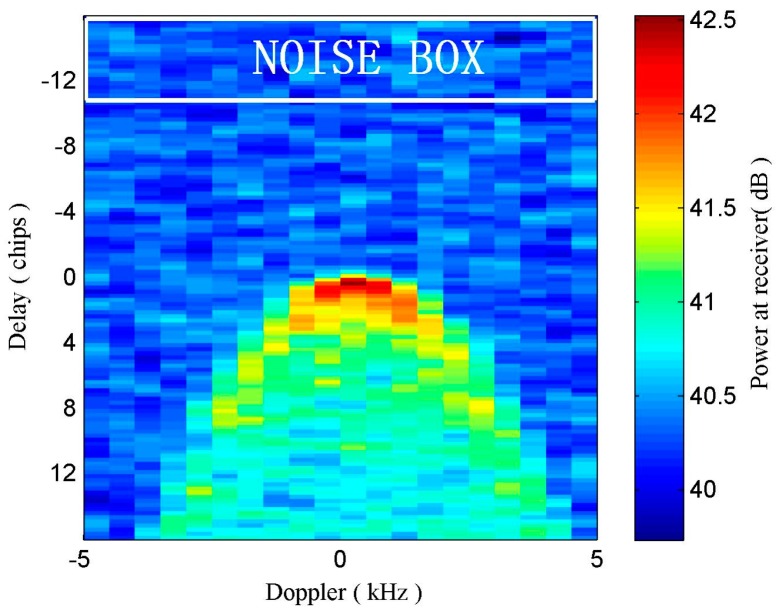
GNSS-R DDM collected over sea water surface and affected by noise. ${\mathrm{DDM}}_{noise}$ denoted as noise floor is the average value of grids within the white box.

Before noise floor subtraction, data alignment is employed to each DDM through adjusting the peak power point to the location without delay and Doppler shift (τ=0, f=0). Note that some DDMs may be seriously affected by noise and cannot demonstrate the characteristics of reflected surface correctly. Thus, DDMs with peak Signal-to-Noise Ratio (SNR) lower than 0 dB are not used. SNR can be calculated as follows:

(7) $$\mathrm{SNR}=\left(DDM(\tau ,f)-DDM_{noise}\right)/DDM_{noise},$$

where DDM(τ,f) is the raw power value of DDM, $DDM_{noise}$ is the noise floor, which can be calculated by:

(8) $$DDM_{noise}=\frac{1}{N}\sum_{\tau_{1}}^{\tau_{m}}\sum_{f_{1}}^{f_{m}}DDM(\tau ,f),$$

where $DDM_{noise}$ represents the noise floor value; $\tau_{1}$ and $\tau_{m}$ are the pixel limits of delay rows of noise box; $f_{1}$ and $f_{m}$ are the pixel limits of Doppler shift of noise box; *N* represents the number of pixels of the noise box. The noise box chosen in this paper spans twenty Doppler bins and five delay chips starting from 1st to 20th in the standard DDM (shown in Figure 2). Then, the DDM values are respectively subtracted by the corresponding noise floor.

#### 2.3.2. Incoherent Averaging and Normalization for Each DDM

Each dataset contains a large number of DDMs and a certain number of consecutive DDMs are associated with the same type of reflected surface. To improve computational efficiency and reduce the impact of speckle noise, incoherent averaging is employed to the DDMs. However, as pointed out in [40], GNSS-R specular point ground track traverses different kinds of surface at some time intervals, and hence one DDM can be related to a transition area with different surface types, such as ice and water. Similar to the idea of Yan [40], an adaptive incoherent averaging is implemented here and a shorter incoherent averaging interval is used for DDMs collected over transition areas. The TDS-1 data contain the specular point positions, so DDMs with specular points near coastline and ice-water transition areas will be processed with a shorter incoherent averaging duration. The sea ice-water transition area can be roughly detected through observing the changing trend of DDM peak power as abrupt power change occurs over sea ice-water transition areas.

**Figure 3:**
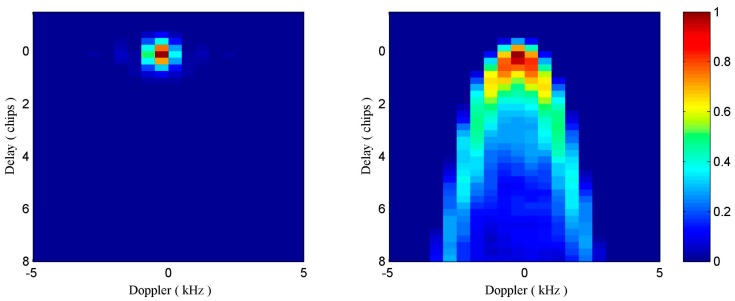
Left plot: Processed GNSS-R DDM collected over sea ice field (middle specular point location: 5°40′23.14″ W, 77°52′1.62″ N, PRN 24) and right plot: Processed GNSS-R DDM collected over sea water field (middle specular point location: 17°44′41.03″ E, 73°42′20.78″ N, PRN 24) by the SGR-ReSI receiver onboard the TDS-1 satellite on 19 February 2015.

After noise reduction and incoherent averaging, normalization is employed to the processed DDMs according to Equation (1). The examples of the resultant DDMs of sea ice and water with 5-s incoherent averaging are shown in Figure 3.

#### 2.3.3. Differential DDM (dDDM) and overall normalization

Let $\bar{DDM_{1}^{i}}\left(\tau ,f\right)$ and $\bar{DDM_{2}^{i}}\left(\tau ,f\right)$ be two adjacent normalized DDMs from sea ice field, let $\bar{DDM_{1}^{w}}\left(\tau ,f\right)$ and $\bar{DDM_{2}^{w}}\left(\tau ,f\right)$ be two adjacent normalized DDMs from sea water field. Then dDDMs between ice and ice, ice and water, water and water can be obtained according to Equation (4). The corresponding equations are shown below:

(9) $$\left\{ \begin{array}{c} {\mathrm{DDM}}_{D}^{ii}\left(\tau ,f\right)\;=\;\bar{DDM_{1}^{i}}(\tau ,f)-\bar{DDM_{2}^{i}}(\tau ,f)\\ {\mathrm{DDM}}_{D}^{iw}\left(\tau ,f\right)\;=\bar{\;DDM_{2}^{i}}(\tau ,f)-\bar{DDM_{1}^{w}}(\tau ,f)\\ {\mathrm{DDM}}_{D}^{ww}\left(\tau ,f\right)\;=\bar{\;DDM_{1}^{w}}(\tau ,f)-\bar{DDM_{2}^{w}}(\tau ,f)\\ {\mathrm{DDM}}_{D}^{wi}\left(\tau ,f\right)\;=\;\bar{DDM_{2}^{w}}(\tau ,f)-\bar{DDM_{1}^{i}}(\tau ,f) \end{array}\right..$$

**Figure 4:**
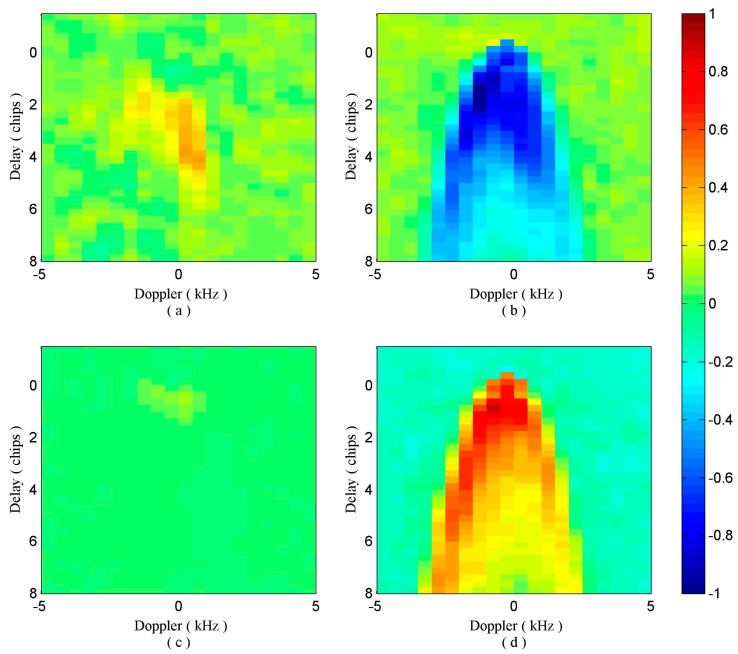
Normalized dDDMs for four different observation situations: (**a**) water-water transition, (**b**) water-ice transition, (**c**) ice-ice transition and (**d**) ice-water transition.

Considering that the values of ${\mathrm{DDM}}_{D}\left(\tau ,f\right)$ are relatively small, we normalized the various dDDMs by ${\left|DDM_{D}\left(\tau ,f\right)\right|}_{\max}$, where ${\left|DDM_{D}\left(\tau ,f\right)\right|}_{\max}$ is the maximum absolute value of obtained dDDMs. Since it is useful to know what ${\mathrm{DDM}}_{D}\left(\tau ,f\right)$ looks like, examples of ${\mathrm{DDM}}_{D}\left(\tau ,f\right)$ associated with the four different scenarios are shown in Figure 4. Clearly, these four pictures are significantly different from each other, providing the basis to identify the transition type. To remain consistency and avoid performance degradation, the same normalization with the same maximum value is applied to all the dDDMs of the specific dataset.

### 2.4. Proposed Identification Approach

**Figure 5:**
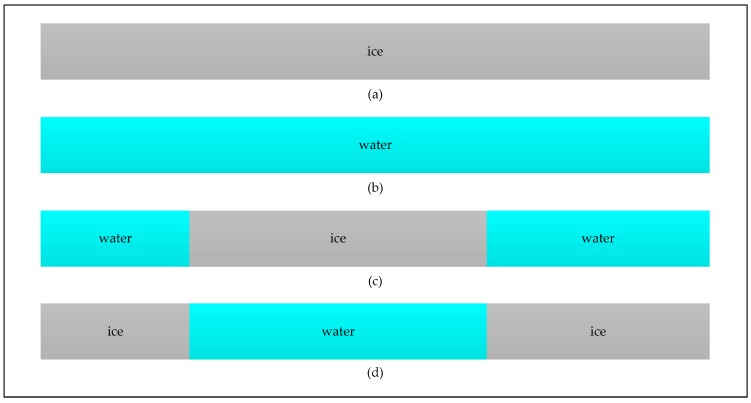
Four scenarios for one dataset which includes: (**a**) DDMs only collected over sea ice field, (**b**) DDMs only collected over sea water field, (**c**,**d**) DDMs collected over both sea ice and water field.

As illustrated in Figure 4, the differential power values of $\bar{{\mathrm{DDM}}_{D}}\left(\tau ,f\right)$ between the same reflected surfaces (Figure 4a,c) basically range from −0.2 to 0.3, while those between different reflected surfaces (Figure 4b,d) have a much wider range and show horseshoe shape. This significant difference provides an opportunity to distinguish dDDMs of different reflected surfaces from those of the same reflected surfaces. Moreover, dDDMs of water-ice transition show a very different power range from those of ice-water transition. This characteristic can be used to distinguish water-ice from ice-water transition. The dDDMs of water-water transition also show different spreading characteristics from those of ice-ice transition. The degree of difference between two adjacent DDMs reflected from ice field is quite small as the surface is suppressed by ice, while dDDMs of water-water transition show larger spreading characteristics than those of ice-ice transition due to the effect of sea surface. The different characteristics can be used to distinguish water-water from ice-ice transition as reflected signals are more sensitive to surface of open water than that of ice covered area. Unfortunately, it seems difficult to build the relationship between the characteristics of dDDMs and the roughness of reflected surface due to the nature of the TDS-1 data (without calibration) and the characteristics of the GPS-R instrument onboard the TDS-1 satellite was not optimized for altimetry purposes. Four scenarios for one dataset which contains a certain number of consecutive DDMs are shown in Figure 5.

#### 2.4.1. Approach Description

The power values of ${\mathrm{DDM}}_{D}\left(\tau ,f\right)$ range from −1 to 1, so a power value threshold ($DDM_{T}$, which ranges from 0 to 1) can be chosen to identify ice-water and water-ice transition. Another power value threshold $DDM_{T}^{\prime }$ ($DDM_{T}^{\prime }\leq DDM_{T}$) is chosen to distinguish ice-ice transition from water-water transition. Two methods are proposed to identify the transition-case, which are presented as follows:

(a) Power Summation of dDDM (PS-D)

The power summation (*PS*) of the grids with absolute values ($\left|\bar{{\mathrm{DDM}}_{D}}\left(\tau ,f\right)\right|$) greater than a preset threshold ($DDM_{T}$) can be obtained using Equation (5). Similarly, the power summation (*PS’*) of the grids with absolute values ($\left|\bar{{\mathrm{DDM}}_{D}}\left(\tau ,f\right)\right|$) greater than a threshold ($DDM_{T}^{\prime }$) can also be obtained using Equation (5). If the power summation threshold for determining ice-water and water-ice transition is defined as $P_{T}$, the threshold for distinguishing ice-ice transition from water-water transition is defined as $P_{T}^{\prime }$, the identification procedure is described as follows:

- If $PS>P_{T}$, two adjacent DDMs are determined as ice-water transition;
- If $PS<-P_{T}$, two adjacent DDMs are determined as water-ice transition;
- If $-P_{T}\leq PS\leq P_{T}$, two adjacent DDMs are determined as the same observing surface and further processing should be done to distinguish between ice-ice and water-water transition:
  ➢ If $\left|PS^{\prime }\right|>P_{T}^{\prime }$, two adjacent DDMs are determined as water-water transition;
  ➢ If $\left|PS^{\prime }\right|\leq P_{T}^{\prime }$, two adjacent DDMs are determined as ice-ice transition.

(b) Pixel Number of dDDM (PN-D)

Similar to PS-D, the abstract pixel number (*PN* and *PN’*) of the grids with absolute values ($\left|\bar{{\mathrm{DDM}}_{D}}\left(\tau ,f\right)\right|$) greater than a preset threshold ($DDM_{T}$ and $DDM_{T}^{\prime }$) of each normalized dDDM can be obtained using Equation (6). If the pixel number threshold for determining ice-water and water-ice transition is defined as $N_{T}$, the threshold for distinguishing between ice-ice and water-water transition is defined as $N_{T}^{\prime }$, the corresponding sea ice detection scheme can be presented as follows:

- If $PN>N_{T}$, two adjacent DDMs are determined as ice-water transition;
- If $PN<-N_{T}$, two adjacent DDMs are determined as water-ice transition;
- If $-N_{T}\leq PN\leq N_{T}$, two adjacent DDMs are determined as the same observing surface and further processing should be done to distinguish between ice-ice and water-water transition:
  ➢ If $\left|PN^{\prime }\right|>N_{T}^{\prime }$, two adjacent DDMs are determined as water-water transition;
  ➢ If $\left|PN^{\prime }\right|\leq N_{T}^{\prime }$, two adjacent DDMs are determined as ice-ice transition.

As illustrated above, water-ice and ice-water transition can be figured out firstly using PS-D and PN-D while the discrimination between ice-ice and water-water transition need further processing. The observing surface types of DDMs from datasets collected over both ice and water field (Figure 5c) can be derived according to ice-water and water-ice transition. DDMs of datasets only collected over ice (Figure 5a) are distinguished from those of datasets only collected over water (Figure 5b) through using similar scheme for determining ice-water and water-ice transition.

#### 2.4.2. Deriving Thresholds

As discussed above, the scenarios can be divided into different-surface (i.e., ice-water and water-ice transition) and same-surface (i.e., ice-ice and water-water transition). For PS-D based method, if the maximum absolute power summation value of same-surface (${\left|PS\right|}_{\max}^{s}$) is always smaller than the minimum absolute value of different-surface (${\left|PS\right|}_{\min}^{d}$), it will be error free to distinguish between different-surface and same-surface. Similarly, if the maximum absolute pixel number value of same-surface (${\left|PN\right|}_{\max}^{s}$) is smaller than the minimum absolute value of different-surface (${\left|PN\right|}_{\min}^{d}$), different-surface and same-surface can be correctly distinguished from each other.

After determining the thresholds for identifying ice-water and water-ice transition, datasets only collected over only ice or water are used to derive the thresholds for distinguishing between ice-ice and water-water transition. Theoretically, if the maximum absolute *PS* and *PN* values of ice-ice transition are always smaller than the minimum absolute values of water-water transition, it will be error free to distinguish ice-ice transition from water-water transition. While the difference between ice-ice transition and water-water transition is much smaller than that between different-surface and same-surface. Therefore, it may be difficult to obtain such error free thresholds. Actually, if more than eighty percent of dDDMs of one dataset collected over only one type of observing surfaces are determined as ice-ice transition, the type of surface of this dataset can be determined as ice.

**Figure 6:**
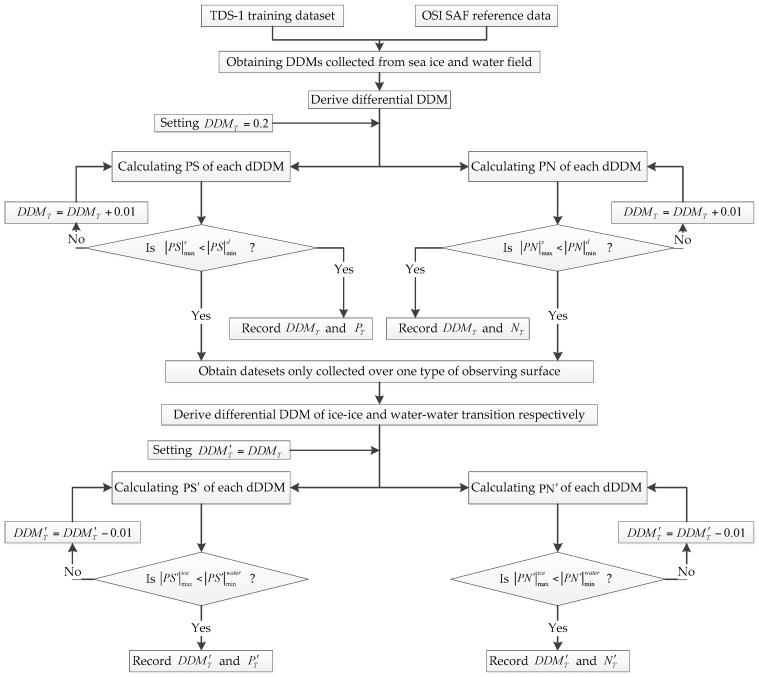
Flowchart of threshold determination for sea ice detection scheme. Six datasets over the Arctic region from TDS-1 are used as training data and sea ice edge data from OSI SAF is used as ground-truth data for determining thresholds.

In order to apply this sea ice detection scheme, six sets of DDM data [46] were used as training data to derive these thresholds so that they may be used as a guideline in the algorithm development. Sea ice edge maps [47] with a resolution of 10 km × 10 km provided by OSI SAF are used as reference data for deriving thresholds. The power value threshold ($DDM_{T}$) for identifying ice-water and water-ice transition is set as 0.2 firstly and increased with a step size of 0.01. Then, different *PS* and *PN* results for each $DDM_{T}$ can be obtained according to PS-D and PN-D respectively. After deriving thresholds for identifying ice-water and water-ice transition, datasets only collected over ice or water field can be identified. Then, the power value threshold ($DDM_{T}^{\prime }$) for distinguishing between ice-ice and water-water transition is set as $DDM_{T}$ ($DDM_{T}=0.2$) firstly and decreased with a step size of 0.01. Then, different *PS’* and *PN’* results for each $DDM_{T}$ can be obtained according to PS-D and PN-D respectively. For PS-D based method, if the maximum absolute *PS* value of more than 85% of ice-ice transition dDDMs (${\left|PS^{\prime }\right|}_{\max}^{ice}$) is smaller than the minimum absolute value of water-water transition (${\left|PS^{\prime }\right|}_{\min}^{water}$), it will provide the basis for distinguishing ice-ice transition from water-water transition. Similarly, if the maximum absolute *PN* value of more than 85% of ice-ice transition dDDMs (${\left|PN^{\prime }\right|}_{\max}^{ice}$) is smaller than the minimum absolute value of water-water transition (${\left|PN^{\prime }\right|}_{\min}^{water}$), ice-ice transition and water-water transition can be correctly distinguished from each other. It was found that $DDM_{T}$ ranging from 0.22 to 0.53 and $DDM_{T}^{\prime }$ ranging from 0.12 to 0.26 can obtain the best detection results. The process of obtaining thresholds is illustrated in Figure 6.

## 3. Experimental Results

### 3.1. Experimental DDM Data Set

The proposed method is evaluated using GNSS-R DDM data produced on TDS-1 when flying over the Arctic region. The Arctic consists of the Arctic Ocean, adjacent seas, and islands. Land within the Arctic region has seasonally varying snow and ice cover, with predominantly treeless permafrost-containing tundra. Arctic seas contain seasonal sea ice in many places. Sea ice of Arctic has a significant effect on global climate and local development, so it is very important to study the ice coverage and its changing trend. The experiments include two parts. Firstly, a case study is implemented through analyzing 131 DDMs from one GNSS-R SP ground track collected on 26 October 2015 in Section 3.2. In Section 3.3, 17346 DDMs from 130 GNSS-R SP ground tracks collected in six separate days from 24 March 2015 to 26 March 2016 have been employed to detect sea ice and water. Meanwhile, the sea ice changing trend over one year can be derived from experimental results.

### 3.2. Case Study

**Figure 7:**
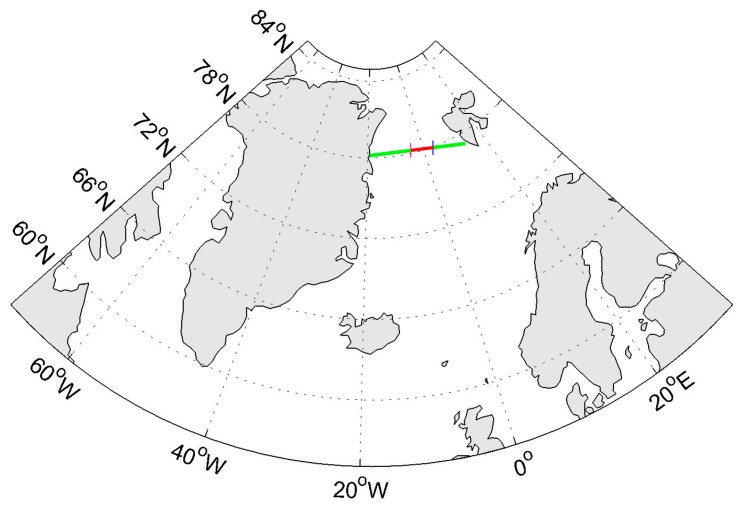
GNSS-R Specular Points (SP) ground-track (green and red line) of TDS-1 signal related to the tested dataset collected over Greenland Sea on 26 October 2015 at 17:29 UTC to 17:31 UTC. The track goes from the East to the West. dDDMs corresponding to SP ground-track in red line are presented in Figure 8. The location of DDM 45 (3°23’35.44” E, 77°57’5.44” N) and DDM 75 (4°26’20.72” W, 78°14’11.77” N) are marked with a blue line and purple line, respectively.

In order to illustrate the proposed sea ice detection methods, a dataset collected over sea ice and water field is selected to depict the detailed process of distinguishing between sea ice and water. Some basic information about tested dataset is shown in Table 1 and the specular points (SP) ground-track is shown in Figure 7.

**Figure 8:**
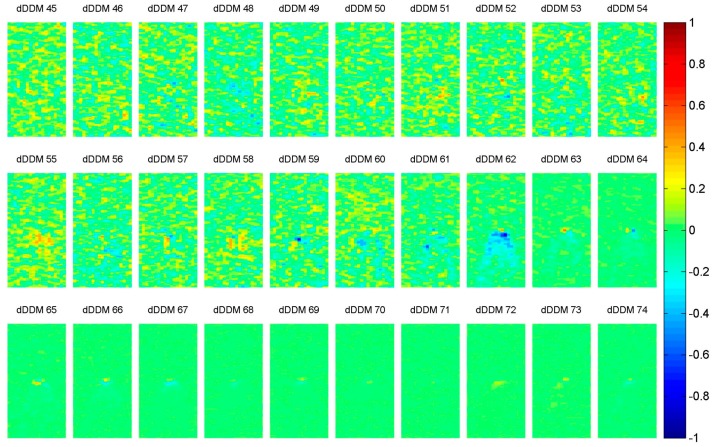
Examples of processed dDDMs (dDDM 45 to dDDM 74) obtained from DDM 45 to DDM 75 of water-water transition, water-ice transition and ice-ice transition.

The sea ice detection scheme is applied to 131 GNSS-R DDMs and hence 130 dDDMs. For illustration purpose, 30 dDDMs (dDDM 45 to dDDM 74) are displayed in Figure 8.

**Figure 9:**
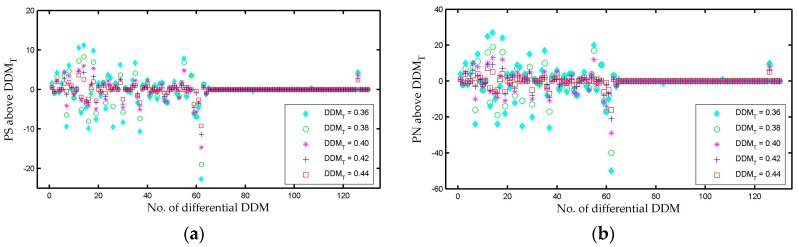
(**a**) PS-D method results and (**b**) PN-D method results for five different thresholds ($DDM_{T}$ = 0.36, $DDM_{T}$ = 0.38, $DDM_{T}$ = 0.40, $DDM_{T}$ = 0.42 and $DDM_{T}$ = 0.44).

The three rows of dDDMs presented in Figure 8 depict three different transition cases, respectively. However, in order to analyze a large amount of data automatically by software, PS-D and PN-D methods described in Section 2.4 are employed to obtained dDDMs. Results of PS-D and PN-D under five different power value thresholds are shown in Figure 9a,b, respectively. Both PS and PN of dDDMs show an abrupt change at dDDM 62 from variation to almost a constant value, so this location provides the basis of determining the boundary between water and sea ice. According to the proposed approach, the first 61 dDDMs are identified as water-water transition, while the 62th dDDM is determined as water-ice transition and the next 67 dDDMs are determined as ice-ice transition. Therefore, the first 62 DDMs were collected over sea water field, while the remaining 69 DDMs were collected over sea ice field.

**Figure 10:**
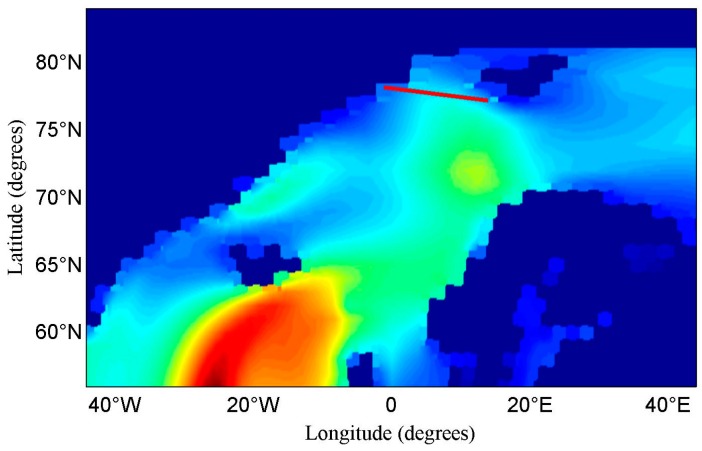
Significant Wave Height data from the European Centre for Medium-Range Weather Forecasts (HW-ECMWF) over high latitude area of the north hemisphere (Latitude: 56° N–84° N, Longitude: 44° W–40° E) on 26 October 2015. The red line is SP ground-track of DDMs (DDM 1 to DDM 57). There is no valid wave height from DDM 58 to DDM 131.

**Figure 11:**
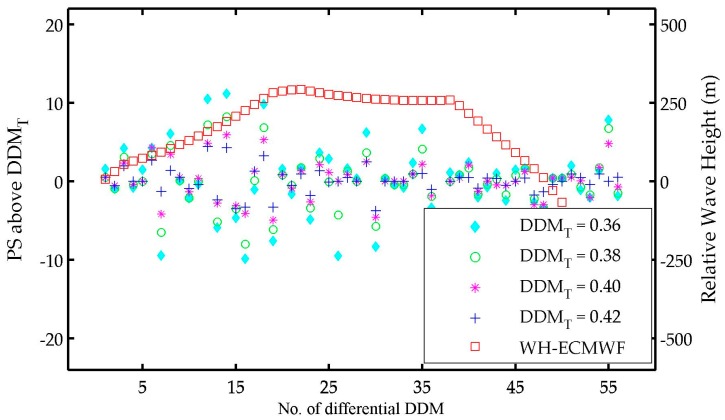
The relationship between PS and WH-ECMWF of dDDM 1 to dDDM 56. Four groups of PS results ($DDM_{T}$ = 0.36, $DDM_{T}$ = 0.38, $DDM_{T}$ = 0.40, $DDM_{T}$ = 0.42) is presented. The values of WH are scaled down as we just concentrate on the variability.

There is a large variability in PS and PN from dDDM 1 to dDDM 37, then a relatively stable variability from dDDM 38 to dDDM 58, and again large change from dDDM 59 to dDDM 61. This phenomenon may be associated with the sea wave height (WH) over the studied area. The significant wave height data (shown in Figure 10) of 26 October 2015 was retrieved from the European Centre for Medium-Range Weather Forecasts (ECMWF, available online: https://www.ecmwf.int) for validating our results. The SP ground-track of 57 DDMs (DDM 1 to DDM 57) is marked in the WH map with a red line, while there is no valid WH data from DDM 58 to DDM 131. The WH of these 57 SP was retrieved through a 2-D linear interpolation according to the SP locations. The values of WH were scaled down as we only concentrate on the variability, which may explain the variability of dDDMs. The relationship between relative wave height and PS of dDDM is presented in Figure 11. The variability of WH can be classified into three stages: (1) the WH changes rapidly from dDDM 1 to dDDM 18 and (2) a relatively slow change from dDDM 19 to dDDM 38 and (3) a decrease from dDDM 38 to dDDM 56. Only the large variability in PS and PN from dDDM 1 to dDDM 18 shows agreement with the changing of WH. Therefore, the variability should be affected by the ocean surface (e.g., wave height, wind speed, sea state etc.), but it is difficult to make any specific conclusions or models about the ocean surface.

### 3.3. Overall Experimental Results

As introduced in Section 3.1, a number of datasets containing a large number of DDMs spanning over twelve months (March 2015, June 2015, October 2015, January 2016 and March 2016) were employed in this section to validate the proposed sea ice detection approach. Only DDMs collected over sea ice and water field are used and DDMs over land are excluded based on the position of specular point and geographic information. Moreover, datasets collected over sea water far from Arctic center region are also excluded.

**Figure 12:**
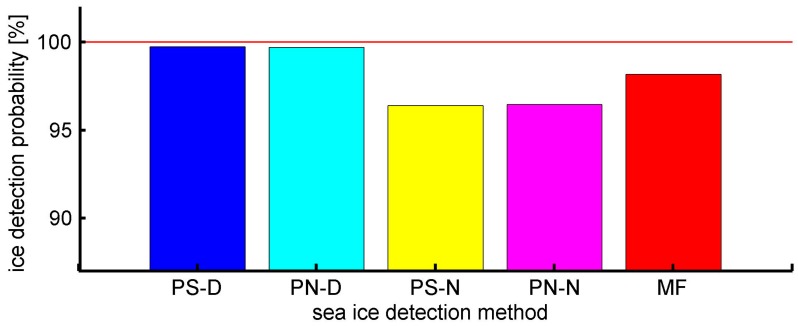
Sea ice detection probability of four different detection methods. PS-D (blue bar) and PN-D (cyan bar) based methods show better performance than PS-N (yellow bar), PN-N (magenta bar) and MF (red bar) based approaches.

In order to assess the performance of the proposed methods, another two observables (power summation of normalized DDM (PS-N) [30], pixel number of normalized DDM (PN-N) [31]) and a matcher filter (MF) approach proposed by Alonso-Arroyo et al. [42] were also used to detect sea ice. Thresholds for best detection results are used for each methods and the probability of false detection of the four methods are presented in Table 2. The sea ice edge data provided by OSI SAF are used as the ground-truth data to examine the detection results. The probability of false detection results for five methods are illustrated in Figure 12. The PS-D and PN-D methods achieve similar performance with a detection probability of 99.72% and 99.69%, respectively, while the detection probabilities of the PS-N, PN-N and MF methods are 96.38%, 96.45% and 98.16%, respectively. Compared to the proposed method PS-D and PN-D, the computation complexity of MF is about 10% lower than that of PS-D and PN-D, but the detection probability of MF is about 1.5% less than that of PS-D and PN-D. The results retrieved by MF show about 97.9% agreement with those obtained by PS-D and PN-D. Moreover, the performance of MF presented in this paper shows good agreement with that presented in [42].

**Figure 13:**
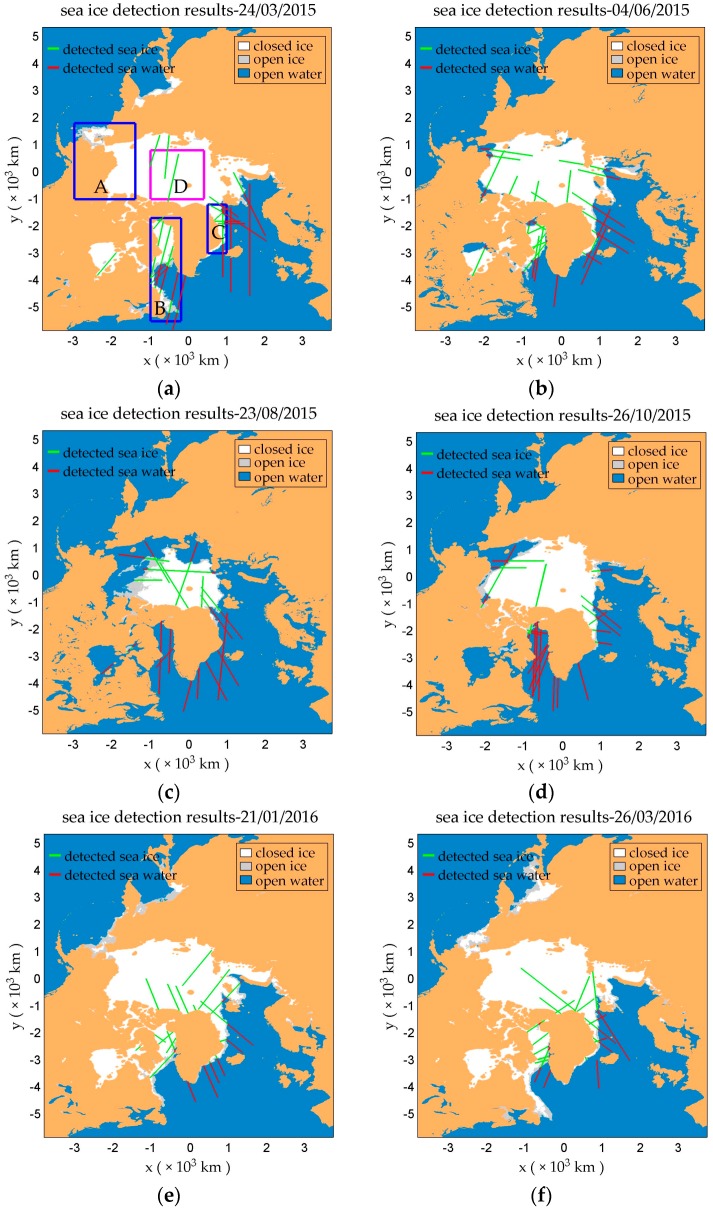
Sea ice detection results based on PS-D using differential and normalized DDM observables in six different days: (**a**) 24 March 2015, (**b**) 4 June 2015, (**c**) 23 August 2015, (**d**) 26 October 2015, (**e**) 21 January 2016, (**f**) 26 March 2016. Lines in green and red represent detected sea ice and water respectively. Three areas marked with blue rectangles are defined as A, B and C respectively. The area marked with magenta rectangle is regarded as D. The white and gray area depicts the ground-truth sea ice coverage (sea ice edge product of the EUMETSAT Ocean and Sea Ice Satellite Application Facility, OSI SAF, www.osi-saf.org).

It is useful and interesting to illustrate sea ice detection results directly on a geographical map. Figure 13 shows the sea ice detection results based on the PS-D method using data collected over six periods (24 March 2015, 4 June 2015, 23 August 2015, 26 October 2015, 21 January 2016 and 26 March 2016).

There was a significant decline in the coverage of sea ice over Arctic region from March to August 2015 due to the increase of temperature. On the other hand, as the temperature decreased from August 2015 to March 2016, the coverage of sea ice increased up to almost the same coverage of March 2015.

As shown in Figure 13a, the change of sea ice coverage mainly happened in the area of A, B and C, which are marked with blue rectangles. Sea ice in these areas can be recognized as young ice or first-year ice which includes both closed ice and open ice, while sea ice in area D marked with magenta rectangle can be regarded as multi-year ice (only include closed ice) [48]. The results show that the sea ice detection probability of closed ice and open ice in the area of A, B and C is 100% and 97.56%, respectively, while the sea ice detection probability of D (multi-year ice) is 98.74%. Generally speaking, first-year ice is relatively smoother [49] and DDMs collected over first-year ice show less spreading, which benefits for sea ice detection. The surface of multi-year ice may be rougher, likely increasing false detection probability.

Although the proposed methods hold great potential for monitoring sea ice, a small quantity of false detection still occurs around the open drift ice and multi-year ice areas. The received signals reflected from both sea ice and water may lead to false detection. Thus, more in-depth research into the relationship between reflected signals and sea ice concentration in needed in the future. Having a good knowledge about sea ice concentration will benefit sea ice detection a lot.

## 4. Conclusions

In this paper, GNSS-R DDM measurements collected by the SGR-ReSI on board the TDS-1 satellite are used for distinguishing between sea ice and water. A dDDM observable is proposed for the first time and two methods (PS-D and PN-D) based on power-summation and pixel-number of dDDMs are proposed to detect sea ice. Meanwhile, several data processing schemes including noise subtraction, incoherent averaging and overall normalization are employed to improve significantly the performance of proposed sea ice detection approaches. The feasibility of PS-D and PN-D methods is validated with ground-truth sea ice edge data provided by OSI SAF and the results indicate the good performance of PS-D and PN-D with a detection probability of 99.72% and 99.69% respectively and a probability of false detection of 0.28% and 0.31% respectively. The proposed methods considerably outperform the existing methods with an improved detection probability of more than 1.5% and significantly reduced probability of false detection.

The dDDMs of water-water transition show larger spreading characteristics than those of ice-ice transition due to the larger variability of reflected surface. This characteristic allows us to distinguish water-water from ice-ice transition, while it does not allow us to model the relationship between received signals and the roughness as the TDS-1 data was not calibrated and the instrument onboard the TDS-1 satellite was not optimized for altimetry purposes.

In the future, it is desirable to further reduce the sea ice false detection probability to obtain more accurate sea ice coverage. Open ice affects sea ice detection and new techniques are needed to handle this issue. Moreover, data coverage rate of TDS-1 is rather limited but CYGNSS launched in 2016 and the potential GEROS-ISS [50] mission will provide much wider coverage.

## Figures and Tables

**Table 1:** Information for the tested dataset.

**Start time**	17:29:01 26 October 2015
**End time**	17:31:11 26 October 2015
**Start point**	13°58′48.05″ E 77°09′55.70″ N
**End point**	19°24′48.51″ W 78°10′31.35″ N
**No. Of DDM**	131
**Ground-track length**	793 km

**Table 2:** Probability of false detection for four observables using GNSS-R DDM.

Date of Dataset	Number of Dataset	Number of Tested DDM	PS-D	PN-D	PS-N	PN-N	MF
24 March 2015	22	2815	0.28	0.36	3.20	3.48	1.88
4 June 2015	25	2906	0.34	0.41	3.99	3.92	1.72
23 August 2015	20	2668	0.22	0.30	3.67	3.37	1.95
26 October 2015	22	3411	0.29	0.23	3.28	3.34	1.91
21 January 2016	21	2764	0.22	0.29	4.12	3.98	1.77
26 March 2016	20	2782	0.29	0.29	3.52	3.24	1.83
Total	130	17346	0.28	0.31	3.62	3.55	1.84
